# Glutamine supplementation moderately affects growth, plasma metabolite and free amino acid patterns in neonatal low birth weight piglets

**DOI:** 10.1017/S0007114522000459

**Published:** 2022-12-28

**Authors:** Zeyang Li, Quentin L. Sciascia, Solvig Görs, Nga Nguyen, Farahnaz Rayatdoost Baghal, Johannes Schregel, Armin Tuchscherer, Jürgen Zentek, Cornelia C. Metges

**Affiliations:** 1 Research Institute for Farm Animal Biology (FBN), Institute of Nutritional Physiology, Dummerstorf, Germany; 2 Research Institute for Farm Animal Biology (FBN), Institute of Genetics and Biometry, Dummerstorf, Germany; 3 Freie Universität Berlin, Department of Veterinary Medicine, Institute of Animal Nutrition, Berlin, Germany

**Keywords:** Birth weight, Piglets, Suckling, Metabolites, Amino acids, Glutamine

## Abstract

Low birth weight (LBW) neonates show impaired growth compared with normal birth weight (NBW) neonates. Glutamine (Gln) supplementation benefits growth of weaning piglets, while the effect on neonates is not sufficiently clear. We examined the effect of neonatal Gln supplementation on piglet growth, milk intake and metabolic parameters. Sow-reared pairs of newborn LBW (0·8–1·2 kg) and NBW (1·4–1·8 kg) male piglets received Gln (1 g/kg body mass (BM)/d; Gln-LBW, Gln-NBW; *n* 24/group) or isonitrogenous alanine (1·22 g/kg BM/d; Ala-LBW; Ala-NBW; *n* 24/group) supplementation at 1–5 or 1–12 d of age (daily in three equal portions at 07:00, 12:00 and 17:00 by syringe feeding). We measured piglet BM, milk intake (1, 11–12 d), plasma metabolite, insulin, amino acid (AA) and liver TAG concentrations (5, 12 d). The Gln-LBW group had higher BM (+7·5%, 10 d, *P* = 0·066; 11–12 d, *P* < 0·05) and milk intake (+14·7%, *P* = 0·015) than Ala-LBW. At 5 d, Ala-LBW group had higher plasma TAG (+34·7%, *P* < 0·1) and lower carnosine (–22·5%, *P* < 0·05) than Ala-NBW and Gln-LBW, and higher liver TAG (+66·9%, *P* = 0·029) than Ala-NBW. At 12 d, plasma urea was higher (+37·5%, *P* < 0·05) with Gln than Ala supplementation. Several proteinogenic AA in plasma were lower (*P* < 0·05) in Ala-NBW *v*. Gln-NBW. Plasma arginine was higher (*P* < 0·05) in Gln-NBW *v* Ala-NBW piglets (5, 12 d). Supplemental Gln moderately improved growth and milk intake and affected lipid metabolism in LBW piglets and AA metabolism in NBW piglets, suggesting effects on intestinal and liver function.

Low birth weight (LBW) is a primary concern for human medicine and animal production^(1)^. Approximately 15–20 % of human infants and 25 % of piglets are born with LBW (birth weight < 2·5 kg and < 1·1 kg, respectively)^(2,3)^. Individuals with LBW show stunted postnatal growth, delayed development and an increased risk of developing insulin resistance and obesity in later life^(4,5)^. Thus, interventions during the early neonatal period designed to improve growth and development may reduce the negative effects associated with LBW.

Glutamine (Gln) is a conditionally indispensable and functional amino acid (AA) that plays an important role in energy and AA metabolism and regulates key metabolic pathways related to growth, immunity and health^(3)^. The neonatal gut grows faster than the other organs^(6)^, and Gln has been reported to be a primary energy source for the neonatal piglet intestine^(7,8)^. Studies investigating Gln supplementation to piglets have mostly focused on the weaning and post-weaning phase^(9)^. Two studies conducted in pre-weaning piglets report positive effects of Gln on growth^(3,10)^. However, the effects of Gln on neonatal LBW piglet growth and milk intake are not sufficiently clear. Gln is a precursor of neurotransmitters in the brain, but there are no reports on whether its supplementation plays a role in the regulation of food intake in piglets^(11,12)^. It is known that piglets that consume more colostrum and milk show better body mass (BM) development^(13,14)^. AA are the building blocks of proteins, are involved in the synthesis of functional molecules (e.g. glutathione, peptide hormones) and regulate metabolic pathways (e.g. pyrimidine and purine precursors; urea synthesis), all of which are related to growth and health^(3,11,15)^. The plasma-free AA pool plays a pivotal role in AA metabolism, as organs and tissues absorb/release free AA from/into the circulation and feed cellular protein metabolism^(16)^. Plasma metabolites such as alanine aminotransferase (ALT) and aspartate aminotransferase (AST) and TAG reflect health and metabolic status of an individual^(17–19)^. However, data on the effect of Gln on the plasma-free AA and metabolite concentrations in neonatal piglets are limited. This information is important, as neonatal piglets are considered an excellent translational model for human infants and children^(20–22)^ and can provide information on whether and how Gln influences early growth and whether it is associated with changes in AA and metabolic patterns in LBW individuals. Our hypothesis was that neonatal Gln supplementation can improve growth and affect plasma metabolite and free AA concentrations in LBW and normal birth weight (NBW) piglets during the neonatal phase. Therefore, this study examined the effect of Gln supplementation on growth, milk intake, plasma metabolites, insulin, free AA and liver TAG concentrations in neonatal LBW and NBW piglets.

## Materials and methods

### Animals and experimental design

All experimental procedures were performed according to the German Animal Welfare Act following the Directive 2010/63/EU (European Convention for the Protection of Vertebrate Animals used for Experimental and Other Scientific Purposes) and approved by the licensing authority State Office for Agriculture, Food Safety and Fishing Mecklenburg-Western Pomerania, Germany (permission No. 7221·3–1–026/16).

**Fig. 1.**
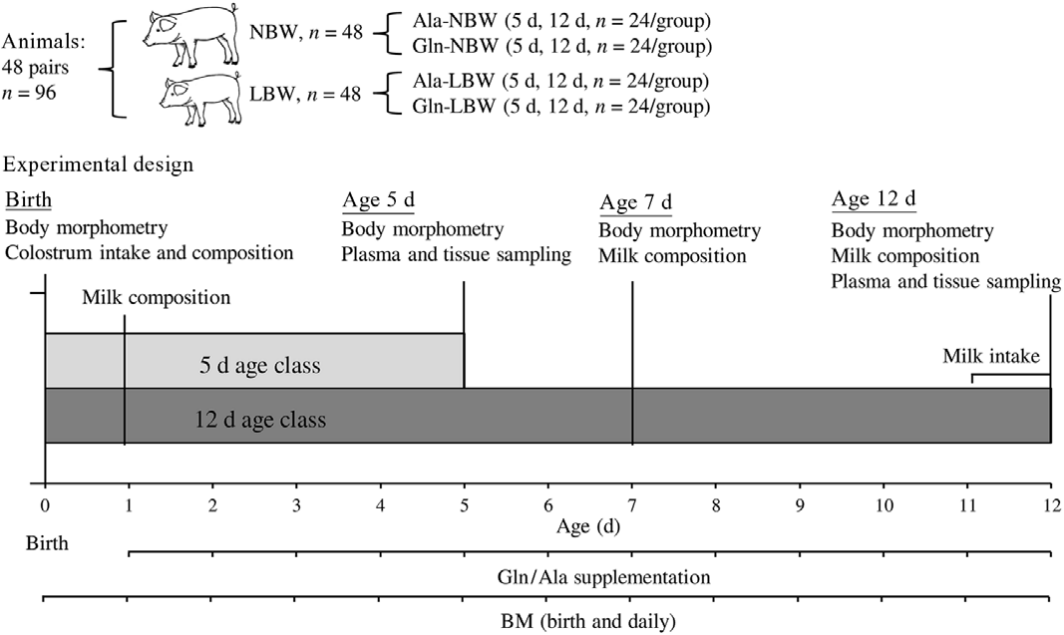
Experimental design showing newborn piglets with low and normal birth weight supplemented with glutamine or alanine starting at age 1 d. Low birth weight (LBW) and normal birth weight (NBW) class piglets were 0·8–1·2 and 1·4–1·8 kg, respectively. At birth, pairs of piglets were allocated to supplemental group (Ala and Gln) and age class (5 d and 12 d). Each age class contained four subgroups (Ala-LBW, Ala-NBW, Gln-LBW and Gln-NBW, *n* 12/group). Supplemental Gln and Ala were dosed at 1 and 1·22 g/kg BM/d, respectively, and in three portions daily (07:00, 12:00 and 17:00) by syringe feeding. Body morphometry measurements include intra-uterine growth restriction score, crown-rump length, abdominal circumference, BMI, ponderal index and rectal temperature. Ala, alanine; Gln, glutamine; BM, body mass.

German Landrace gilts were bred at the experimental pig facility of the Research Institute for Farm Animal Biology (FBN), Dummerstorf, Germany, under standard procedures regarding insemination, housing and farrowing^(23)^. Healthy gilts were fed standard pregnancy and lactation diets (pregnancy: 11·4 MJ of metabolisable energy (ME)/kg, 12·6 % crude protein (CP), 3·8 % ether extract (EE), 9 % fibre; lactation: 13·2 MJ of ME/kg, 16·5 % CP, 6 % EE, 5·3 % fibre; Trede & v. Pein), following German energy and nutrient recommendations for pregnant and lactating sows^(24)^. At birth (0 d), 48 pairs of male LBW (0·8–1·2 kg; *n* 48; below the lowest birth weight quartile of the FBN experimental pig herd)^(23)^ and NBW (1·4–1·8 kg; *n* 48; birth weight control) siblings were selected from 30 litters with 10–20 piglets per litter (15·4 ± 0·4 piglets/litter). Only males were chosen to remove sex-specific effects, and it has been reported that males have a higher pre-weaning mortality^(25,26)^; thus, we expected them to benefit more from a potential Gln effect. After farrowing (0 d), the littermate pairs of LBW and NBW piglets were allocated to either the Gln (1 g/kg BM/d; Gln-LBW, Gln-NBW, *n* 24/group) or alanine (Ala) (1·22 g/kg BM/d; isonitrogenous to Gln; Ala-LBW, and Ala-NBW, *n* 24/group) supplementation groups. The assignment of the pairs of piglets was based on their birth weight to ensure that there was no significant difference in the mean birth weight between the supplementation groups (Ala-LBW *v*. Gln-LBW, Ala-NBW *v*. Gln-NBW). The experimental unit was the piglet. The study was conducted across seventeen experimental runs with half of the pairs sampled at 5 d and the other half at 12 d of age (*n* 12/group per supplementation group and age class) (Fig. 1). Five and 12 d were selected because positive allometric growth of the gastrointestinal tract occurs in this early life period fuelled only by the intake of colostrum/milk^(6)^. Litters were standardised to twelve piglets within 24 h of farrowing. Piglets which lost BM for more than 2 consecutive days, showed sickness behaviour or lack of mobility, were excluded from the experiment together with its pair mate. Based on these exclusion criteria, five pairs of LBW and NBW piglets were excluded during the experiment and replaced with matching pairs of replacement piglets to achieve the planned sample size (total *n* 96). Data from all piglets were included in the final data analysis. Experimental staff was not blinded to the supplementation treatment. The supplemental AA was freshly dissolved in water and administered daily in three equal portions (07:00, 12:00 and 17:00) with syringe feeding from 1 to 5 or 1 to 12 d of age (2 ml of water/dose followed by another 2 ml of water to rinse the syringe to ensure the entire AA dose was consumed by the piglets). Dosage times were selected based on our pre-trial data, which showed that oral Gln supplementation increased plasma Gln concentrations in neonatal piglets with a Gln peak (1·06 mmol/l) at 45-min post-supplementation and returned to baseline levels at 4 h (613 µmol/l). The dose of supplemental Gln was equivalent to 59 % of daily Gln intake from sows’ milk at the age of 11–12 d in the present study. Water was offered *ad libitum* to sows and piglets. Room temperature was kept at 20°C, and the pens were equipped with heating lamps for the piglets.

At 5 and 12 d of age, the experimental piglets were transferred to the FBN slaughterhouse together with age-matched non-experimental piglets to reduce stress. The piglets received 33 % of their respective daily AA supplement and 6 ml of milk replacer/piglet (150 g/l water; 20·5 % CP, 10 % EE, 0·2 % fibre; Neopigg Rescuemilk 2·0, Provimi) from 08:00 with a 30-min interval for each subsequent piglet. Two hours after supplementation (10:00 and at 30-min intervals thereafter), the piglets were stunned by bolt gun (5 d) or electro-stunned (12 d) and then euthanised via exsanguination.

### Piglet growth, morphometry and litter characteristics

At birth, piglet sex and birth weight were recorded, and the corresponding birth order numbers were marked on the back of each individual piglet to determine the time to first suckle (first suckle time – birth time, min). Piglet intra-uterine growth restriction (IUGR) score^(25)^ and morphometry including crown-rump length (CRL)^(27)^, abdominal circumference (ACF)^(28)^, BMI^(27)^, ponderal index^(27)^ and rectal temperature were determined at birth, 5, 7 and 12 d. Piglet BM was measured daily. Additionally, the live, dead and mummified piglets per litter were also determined.

### Colostrum/milk composition and piglet intake

Colostrum samples were collected by hand-stripping from the anterior teats (first and second teat pairs) and posterior teats (sixth and seventh teat pairs) within 2 h following birth of the first piglet. Milk was collected at 24 h, 7 and 12 d postpartum 5–10 min after an intramuscular injection of 1 ml of oxytocin (Longacton®, carbetocine, IDT Biologika). Equal amounts (1–3 ml) of individual colostrum/milk from the anterior and posterior teats were pooled and stored at −20°C. Milk DM, EE, CP, lactose and Ig (IgA, IgM and IgG) were determined for each pooled sample (online Supplementary Table 1)^(23,29)^. The CV within and between assays for milk DM, EE, CP, lactose and immunoglobulins were 0·7 % and 1·5 %, 2·6 % and 2·3 %, 5·1 % and 1·4 %, 5·1 % and 6·4 %, and 4·5 % and 6·2 %, respectively. For free AA analysis, samples were prepared as for lactose measurement^(23)^ and analysed using HPLC^(30)^ but with a different column (250 × 4 mm Hyperclone ODS (C18) 120Å column, Phenomenex). Concentrations of protein-bound AA were determined by HPLC after enzymatic protein hydrolysis^(10,31)^, as standard acid hydrolysis converts Gln and asparagine (Asn) to their acid counterparts. The intra- and inter-assay CV for free AA and protein-bound AA were 4·7 % and 5·4 %, and 3·8 % and 7·2 %, respectively.

Colostrum intake was calculated for 24 h after birth based on BM gain (BMG)^(27)^. Milk intake was measured from 11 to 12 d using the deuterium oxide (^2^H_2_O) isotope dilution method^(32)^. Piglets received an intraperitoneal injection of ^2^H_2_O (0·2 ml/kg BM, 70 atom % ^2^H diluted to 20 % in physiological saline) 24 h before euthanasia, followed by isolation (1 h) to prevent suckling and ensure that the ^2^H_2_O had equilibrated with the body water pool. Blood samples were taken 1 h (11 d) and 24 h (12 d, after euthanasia) following ^2^H_2_O injection. In a blood sample from a non-experimental littermate piglet, the background level of ^2^H enrichment was determined. ^2^H enrichment was measured as previously described^(33)^ and used to calculate milk intake^(32,34)^. The CV within and between assays for ^2^H measurements were both < 1 %. Colostrum/milk intake of each individual piglet was multiplied by the mean Gln content in their dams’ colostrum (mean of 2 h and 24 h after birth) or milk (12 d) to calculate the Gln intake from sows’ colostrum/milk.

### Plasma metabolite, insulin and free amino acid concentrations

Blood samples were collected at euthanasia via cardiac puncture in K-EDTA tubes (Sarstedt), centrifuged at 1576 *g* for 20 min (4°C). The resulting plasma was stored at −80°C for further analysis. The activities of ALT and AST and the concentrations of albumin, bilirubin, cholesterol, glucose, urea, lactate, NEFA, total protein and TAG in plasma were measured^(35,36)^. The CV within and between assays for these measurements were < 2·9 % and < 8 %, respectively. Plasma-free AA concentrations were determined using HPLC as described for milk-free AA concentrations. The within- and between-assay CV for free AA were 4·7 % and 5·4 %, respectively. The plasma insulin concentration was analysed by ELISA assay (EIA-4747, DRG Instruments) according to the manufacturer’s instructions with a within and between CV of 2·3 % and 4·4 %, respectively.

### Liver TAG concentrations

At euthanasia, liver samples were collected at the intersection between the left and right medial lobes, avoiding major veins. Liver samples were rinsed with 0·9 % physiological saline, cut into small pieces on a chilled cutting block, immediately frozen in liquid N_2_ and then stored at −80°C. Liver TAG concentrations were determined using the TAG Quantification kit (MAK266, Sigma-Aldrich) according to the manufacturer’s instructions. The CV within and between assays were 5·2 % and 9·5 %, respectively.

### Statistical analysis

The required sample size was calculated with CADEMO for Windows ANOV-version 4.03 (2000; BioMath GmbH). We applied the first kind risk *α* = 0·05, a limit *β* = 0·20 for the second kind risk *β* (i.e. a power of 1 – *β* of at least 0·80) and the minimum difference between the main effects levels d to be detected, which was chosen as a relative c-fold of the residual standard deviation of each parameter. The normality of residuals was tested using the Shapiro–Wilks test in SAS (version 9.4; SAS Institute Incorporated). Data were analysed by ANOVA using the MIXED procedure of SAS or the repeated-measures ANOVA where applicable. Model selection was based on Akaike’s information criterion^(37)^.

Model 1 for the evaluation of BMG, IUGR score, colostrum intake, time to first suckle, milk intake and Gln intake from colostrum/milk contained the fixed effects supplementation (Gln and Ala), birth weight class (BC) (LBW and NBW), the supplementation × BC interaction and a random sow effect. Model 2 (repeated-measures ANOVA) for CRL, ACF, BMI, ponderal index and rectal temperature contained the fixed effects supplementation (Gln and Ala), BC (LBW and NBW), age (day 0, 5, 7 and 12), the supplementation × BC interaction and a random sow effect. Repeated measurements on the same animal at different ages were considered by the repeated statement of the MIXED procedure using the SUBJECT = animal option to define the blocks of the block diagonal residual covariance matrix and the TYPE = UN option to define their unstructured covariance structure. Model 3 (repeated-measures ANOVA) for BM contained the fixed effects supplementation (Gln and Ala), BC (LBW and NBW), age (day 0–12), the interaction supplementation × BC and a random sow effect. Repeated daily measurements on the same animal were considered in the same way as in model 2 but with autoregressive (1) block diagonal residual covariance matrix. The concentrations of plasma metabolites, free AA, insulin and liver TAG were analysed with model 1, which additionally contained the fixed factor experimental run. Given the age-dependent difference in development in piglets, we conducted the analyses separately for 5- and 12-d-old piglets. Model 4 (repeated-measures ANOVA) was used for milk composition and contained the fixed effects stage of lactation (2 h, 24 h, 7 d and 12 d), teat number (1 + 2, 6 + 7), litter mass (total born piglets’ classes: 10–13, 14–17 and > 17) and experimental run. The stage of lactation and teat number were repeated factors, and the block diagonal residual covariance matrix structure was UN@CS, that is, the direct product of the (4 × 4) unstructured matrix of the lactation stages and the (2 × 2) matrix of the teat number having a compound symmetry structure. Least-squares means and their standard errors were computed for each fixed effect in the models. The SLICE statement was used for performing partitioned analyses of the least-squares means for the supplementation × BC or supplementation × BC × age interactions. Pearson correlations were computed between the plasma parameters and liver TAG concentrations or BM of piglets at 5 and 12 d of age. Differences were considered significant if *P* < 0·05 and a trend was considered if 0·05 ≤ *P* < 0·10 (Tukey–Kramer test).

## Results

### Growth performance and milk intake

Factor supplementation did not affect piglet BM, morphometry or BMG (*P* > 0·1) with the exception of ACF (*P* = 0·077) (Table 1). The BC influenced all growth-related parameters (*P* < 0·05). In Gln-LBW compared with Ala-LBW, piglet BM tended to be higher at 10 d (*P* = 0·066) and was higher at 11 and 12 d of age (*P* < 0·05), ACF was greater (5 d, *P* < 0·01) and CRL tended to be greater (12 d, *P* = 0·051), while BMG did not differ. No differences were observed in piglet BM, morphometry or BMG between Gln-NBW and Ala-NBW piglets.

**Table 1.** Body mass, body morphometry, body mass gain, and milk intake of low and normal birth weight piglets supplemented with glutamine or alanine from age 1 to 12 d

	Ala				Gln				ANOVA *P* ||		
	LBW	se	NBW	se	LBW	se	NBW	se	Sup	BC	Interaction
BM (kg)									0·264	< 0·001	0·580
Birth	1·10†	0·06	1·49	0·06	1·09†	0·06	1·48	0·06			
5 d	1·71†	0·06	2·27	0·06	1·76†	0·06	2·27	0·06			
7 d	2·17†	0·06	2·74	0·06	2·19†	0·06	2·82	0·06			
10 d	2·63†	0·07	3·32	0·07	2·79†,‡	0·07	3·41	0·07			
11 d	2·87†	0·07	3·60	0·07	3·08*,†	0·07	3·69	0·07			
12 d	3·07†	0·07	3·84	0·07	3·30*,†	0·07	3·89	0·07			
CRL (cm)									0·274	< 0·001	0·402
5 d	25·4†	0·6	28·6	0·6	26·4†	0·5	28·5	0·5			
7 d	29·6†	0·6	31·9	0·6	29·8†	0·6	32·2	0·6			
12 d	31·4†	0·6	34·0	0·6	32·9†,‡	0·6	34·3	0·6			
ACF (cm)									0·077	< 0·001	0·921
5 d	26·8†	0·5	29·9	0·5	28·7*,†	0·5	30·3	0·5			
7 d	27·9†	0·6	29·6	0·6	28·1†	0·6	30·3	0·6			
12 d	32·4†	0·6	34·1	0·6	32·4†	0·6	35·4	0·6			
BMI (kg/m2)									0·525	< 0·001	0·925
5 d	25·8	1·0	27·5	1·0	24·9†	1·0	27·9	1·0			
7 d	25·4†	1·0	27·8	1·0	24·9	1·0	27·2	1·0			
12 d	31·9	1·0	34·1	1·0	30·9	1·0	33·3	1·0			
BMG (g/d)											
0–5 d	122†	7·9	156	7·9	129†	7·7	156	7·7	0·610	< 0·001	0·540
6–12 d	192†	13·7	229	13·7	214	13·2	225	13·2	0·400	0·011	0·150
Milk intake (g/kg BM)	328	13·4	296	13·4	376*,†	13·4	335‡	13·4	0·003	0·011	0·730
Gln intake from milk (g/kg BM)	1·57	0·07	1·41	0·07	1·81*,§	0·07	1·62*	0·07	0·003	0·015	0·767

Ala, alanine; Gln, glutamine; LBW, low birth weight; NBW, normal birth weight; Sup, supplementation; BC, birth weight class; BM, body mass; CRL, crown-rump length; ACF, abdominal circumference; BMG, body mass gain.

Values are least-squares means and standard error; BM, *n* 24/group (0–5 d), *n* 12/group (6–12 d); BMG, *n* 24/group (0–5 d), *n* 12/group (6–12 d); milk intake, *n* 12/group; CRL, ACF and BMI; *n* 12/group (5, 7, 12 d).

* Different from Ala-supplemented piglets within BC group (*P* < 0·05).

† Different from NBW piglets within supplemental group (*P* < 0·05).

‡ Tend to differ from Ala-supplemented piglets within BC group (*P* < 0·1).

§ Tend to differ from NBW piglets within supplemental group (*P* < 0·1).

|| ANOVA *F* test.

At birth, LBW piglets had lower birth weight (1·10 ± 0·04 *v*. 1·49 ± 0·04 kg, *P* < 0·001), CRL (22·1 ± 0·3 *v*. 24·3 ± 0·3 cm, *P* < 0·001), ACF (21·9 ± 0·3 *v*. 24·5 ± 0·3 cm, *P* < 0·001), BMI (21·6 ± 0·6 *v*. 24·0 ± 0·6 kg/m^2^, *P* = 0·006) and a higher IUGR score (0·7 ± 0·1 *v*. 0·2 ± 0·1, *P* < 0·001) than NBW piglets. Within each supplemental group, LBW piglets had lower (*P* < 0·05) BM (0–12 d of age), CRL (5, 7 and 12 d of age), ACF (5, 7 and 12 d of age), and BMI (Gln: 5 d, Ala: 7 d of age) than NBW piglets. Piglets’ BMG (0–5 d) was lower (*P* < 0·01) in LBW compared with NBW within each supplemental group, while BMG during 6–12 d was only lower (*P* = 0·006) in Ala-LBW compared with Ala-NBW piglets.

Colostrum intake (146·9 ± 20·7 g/kg BM, first 24 h) and Gln intake from sow colostrum (1·5 ± 0·2 g/kg BM, first 24 h) did not differ between LBW and NBW piglets, even though the time to first suckle tended to be longer in LBW compared with NBW piglets (55·1 ± 10·9 *v*. 40·1 ± 11·0 min, *P* < 0·1). Supplementation and BC affected milk intake (11–12 d of age) and the corresponding Gln intake from milk (*P* < 0·05) (Table 1). Milk intake was higher (*P* = 0·015) in Gln-LBW piglets than in Ala-LBW piglets and tended to be higher (*P* < 0·1) in Gln-NBW than in Ala-NBW piglets. In the Gln-supplemented groups, milk intake was higher (*P* < 0·05) in LBW piglets than in NBW piglets, but it did not differ between the Ala-supplemented groups. Similarly, Gln intake from sow’s milk (11 to 12 d of age) was higher (*P* < 0·05) in Gln- compared with Ala-supplemented piglets in each BC group. Milk Gln intake tended to be higher (*P* < 0·1) in Gln-LBW piglets than in Gln-NBW piglets, while no difference in Ala intake was observed between Ala-LBW and Ala-NBW piglets.

### Plasma metabolite, insulin and liver TAG concentrations

At 5 d of age, the effects of supplementation and the supplementation × BC interaction on piglet plasma metabolites, insulin and liver TAG concentrations were not significant (Table 2). There was a trend towards higher plasma NEFA (*P* = 0·058) and lower plasma TAG (*P* = 0·051) concentrations in Gln-LBW compared with Ala-LBW piglets. The BC affected (*P* < 0·05) plasma ALT, TAG, insulin, liver TAG concentrations and plasma glucose:insulin ratio, with lower plasma glucose:insulin ratio (*P* = 0·034) and a trend towards higher ALT activity (*P* = 0·060) in Gln-LBW *v*. Gln-NBW. The plasma and liver TAG concentrations were higher (*P* < 0·05) and plasma insulin concentrations tended towards higher (*P* = 0·064) values in Ala-LBW *v*. Ala-NBW.

**Table 2.** Concentration of plasma metabolites, insulin and liver TAG at age 5 and 12 d of low and normal birth weight piglets supplemented with glutamine or alanine starting at age 1 d

	Ala				Gln				ANOVA *P* ||		
	LBW	se	NBW	se	LBW	se	NBW	se	Sup	BC	Interaction
Age 5 d											
Plasma											
ALT (U/L)	46·9	2·6	43·0	2·6	47·1§	2·5	41·8	2·5	0·812	0·022	0·728
NEFA (μmol/l)	299·4	25·1	278·9	25·1	363·4† ‡	24·7	297·1	24·7	0·102	0·050	0·291
TAG (mmol/l)	1·2†	0·1	0·8	0·09	0·9‡	0·1	0·9	0·1	0·228	0·016	0·111
Urea (mmol/l)	2·9	0·3	2·9	0·3	3·3	0·3	3·1	0·3	0·245	0·737	0·630
Glucose (mmol/l)	7·5	0·2	7·7	0·2	7·6	0·2	7·7	0·2	0·849	0·208	0·846
Insulin (μU/ml)	5·1§	0·6	3·7	0·6	5·0	0·6	3·9	0·6	0·999	0·022	0·765
Glucose:insulin ratio	0·4	0·1	0·5	0·1	0·3†	0·1	0·5	0·1	0·430	0·014	0·599
Liver											
TAG (mg/g tissue)	25·7†	6·1	15·4	6·2	20·2	6·1	16·2	6·2	0·571	0·033	0·322
Age 12 d											
Plasma											
ALT (U/L)	32·9	1·5	33·6	1·5	35·0	1·6	37·4‡	1·6	0·068	0·314	0·569
NEFA (μmol/l)	361·9	26·3	332·4	26·3	401·5†	26·6	309·4	26·6	0·759	0·025	0·236
TAG (mmol/l)	0·8	0·1	0·7	0·1	0·8	0·1	0·8	0·1	0·333	0·523	0·699
Urea (mmol/l)	2·4	0·3	2·0	0·3	3·2*	0·3	2·8*	0·3	0·006	0·075	0·756
Glucose (mmol/l)	7·6	0·2	8·0	0·2	8·0	0·2	7·8	0·2	0·503	0·532	0·069
Insulin (μU/ml)	6·9†	0·8	4·5	0·8	6·1	0·7	5·3	0·7	0·982	0·022	0·267
Glucose:insulin ratio	0·2	0·1	0·4	0·1	0·2	0·1	0·3	0·1	0·891	0·012	0·693
Liver											
TAG (mg/g tissue)	7·6†	0·8	8·7	0·8	8·0	0·8	7·1	0·8	0·465	0·946	0·155

Ala, alanine; Gln, glutamine; LBW, low birth weight; NBW, normal birth weight; Sup, supplementation; BC, birth weight class; ALT, alanine aminotransferase.

Values are least-squares means and standard error; *n* 12/group (5, 12 d). Plasma samples were obtained at 2 h post-oral administration of Gln or Ala and subsequent milk replacer. Only plasma or liver measurements affected by supplementation or BC at age 5 or 12 d are shown.

* Different from Ala-supplemented piglets within BC group (*P* < 0·05).

† Different from NBW piglets within supplemental group (*P* < 0·05).

‡ Tend to differ from Ala-supplemented piglets within BC group (*P* < 0·1).

§ Tend to differ from NBW piglets within supplemental group (*P* < 0·1).

|| ANOVA *F* test. The factor experimental run was significant for plasma ALT at age 12 d (*P* < 0·001).

At 12 d of age (Table 2), only plasma urea concentration was affected by supplementation (*P* = 0·006), with higher (*P* < 0·05) plasma urea concentrations observed in Gln compared with Ala piglets within both BC. The factor BC was significant (*P* < 0·05) for plasma NEFA, insulin concentrations and glucose:insulin ratio, with higher plasma NEFA concentrations (*P* = 0·017) observed in Gln-LBW *v*. Gln-NBW piglets irrespective of age, and higher plasma insulin concentrations (*P* = 0·018) observed in Ala-LBW *v*. Ala-NBW piglets. The glucose:insulin ratio was lower (*P* = 0·038) in Ala-LBW *v*. Ala-NBW piglets. Plasma glucose concentrations were affected neither by the main factors nor by the supplementation × BC interaction at 5 and 12 d of age.

### Plasma-free amino acid concentrations

The concentration of several plasma-free AA was affected (*P* < 0·05) in 5-d-old piglets by factor supplementation (Table 3). Within the LBW group, Gln-supplemented piglets had higher plasma Gln (*P* = 0·006), a trend towards higher (*P* = 0·081) arginine (Arg) concentrations and lower (*P* < 0·01) Ala and glucogenic amino acids (GAA) concentrations than Ala-supplemented piglets. The NBW piglets supplemented with Gln had higher (*P* < 0·01) plasma Gln and Arg concentrations, whereas the plasma concentrations of Ala, glycine (Gly), dispensable amino acids (DAA) and GAA were lower (*P* < 0·05) than in the Ala piglets. Additionally, plasma carnosine (Car) was affected by supplementation (*P* = 0·015) and the supplementation × BC interaction was significant (*P* = 0·005) for 3-methylhistidine (3-MH) concentration, and Car and 3-MH were higher (*P* < 0·05) in Gln-LBW *v*. Ala-LBW (Table 3). The BC affected (*P* < 0·05) several plasma-free AA concentrations. Within Gln groups, LBW piglets had lower (*P* < 0·05) plasma tryptophan (Trp) and cysteine (Cys) concentrations and a trend towards higher (*P* = 0·061) plasma glutamate (Glu) and lower (*P* = 0·081) plasma Gly concentrations than NBW piglets (Table 3). In LBW *v*. NBW piglets, Ala supplementation reduced plasma Gly, Car, hydroxyproline, taurine (Tau), 1-methylhistidine (1-MH) and 3-MH concentrations (*P* < 0·05), whilst plasma Trp and GAA concentrations tended to be lower (*P* < 0·1). Plasma *γ*-aminobutyric acid (GABA) and *β*-Ala did not differ among groups (Table 3).

**Table 3.** Concentration of plasma-free amino acids at age 5 d of low and normal birth weight piglets supplemented with glutamine or alanine starting at age 1 d

	Ala				Gln				ANOVA *P* ||		
	LBW	se	NBW	se	LBW	se	NBW	se	Sup	BC	Interaction
IAA (μmol/l)											
Arg	117·8	12·5	121·4	12·58	144·4‡	12·3	165·0*	12·3	0·004	0·204	0·368
Trp	32·2§	2·4	35·8	2·4	33·2†	2·4	39·7	2·4	0·197	0·002	0·329
* β*-Ala	22·4	2·3	22·3	2·3	21·5	2·3	26·3	2·3	0·484	0·219	0·198
DAA (μmol/L)											
Ala	1389·2	100·9	1430·3	100·9	833·2*	99·2	779·4*	99·2	< 0·001	0·938	0·560
Cys	102·4	7·8	109·1	7·8	94·3†	7·7	109·2	7·7	0·496	0·023	0·369
Gln	697·3	56·1	658·7	56·1	921·7*	55·8	872·9*	55·3	< 0·001	0·408	0·923
Glu	233·1	15·4	204·1	15·4	248·5§	15·1	213·3	15·1	0·412	0·018	0·809
Gly	661·6†	64·7	877·8	64·7	625·9§	64·0	723·4*	64·0	0·061	< 0·001	0·131
Amino metabolites (μmol/l)											
Car	13·6†	1·2	17·5	1·2	17·6*	1·2	19·3	1·2	0·022	0·015	0·324
Hyp	263·5†	19·7	321·8	19·7	273·7	19·3	282·2	19·3	0·464	0·077	0·183
Tau	105·0†	10·7	138·0	10·7	119·8	10·6	135·8	10·6	0·557	0·012	0·361
1-MH	3·7†	0·7	5·7	0·7	4·6	0·6	5·3	0·6	0·699	0·015	0·264
3-MH	6·8†	0·7	10·2	0·7	9·2*	0·6	8·9	0·6	0·431	0·020	0·005
GABA	1·4	0·1	1·5	0·1	1·3	0·1	1·5	0·1	0·848	0·259	0·897
Groups of AA (mmol/l)											
DAA	5·8	0·32	5·9	0·3	5·2	0·3	5·0*	0·3	0·011	0·753	0·462
GAA	2·4§	0·2	2·7	0·2	1·8*	0·2	1·8*	0·2	< 0·001	0·170	0·260

Ala, alanine; Gln, glutamine; LBW, low birth weight; NBW, normal birth weight; Sup, supplementation; BC, birth weight class; IAA, indispensable amino acid; Arg, arginine; DAA, dispensable amino acid; Car, carnosine; Hyp, hydroxyproline; Tau, taurine; 1-MH, 1-methylhistidine; 3-MH, 3-methylhistidine; GABA, *γ*-aminobutyric acid; AA, amino acid; GAA, glucogenic amino acid; Cys, cysteine.

Values are least-squares means and standard error; *n* 12/group. Plasma samples were obtained at 2 h post-oral administration of Gln or Ala and subsequent milk replacer. Only plasma AA influenced by supplementation or BC are shown.

* Different from Ala-supplemented piglets within BC group (*P* < 0·05).

† Different from NBW piglets within supplemental group (*P* < 0·05).

‡ Tend to differ from Ala-supplemented piglets within BC group (*P* < 0·1).

§ Tend to differ from NBW piglets within supplemental group (*P* < 0·1).

|| ANOVA *F* test. The factor experimental run was significant for plasma 3-MH and Gln (*P* = 0·037).

At 12 d of age, the factors supplementation and supplementation × BC were significant (*P* < 0·05) for the concentrations of several plasma-free AA and groups of AA (Table 4). Within the LBW group, Gln piglets had higher plasma Gln concentration (*P* = 0·010) and lower (*P* < 0·05) Ala, Asn, DAA, GAA, indispensable amino acid (IAA) and total AA concentrations and a trend towards lower (*P* = 0·063) plasma Gly concentrations than Ala piglets. Within the NBW group, plasma Arg, histidine (His), leucine (Leu), methionine (Met), Gln and ketogenic amino acids (KAA) concentrations were higher (*P* < 0·05), plasma Ala concentrations were lower (*P* = 0·001), and lysine (Lys) and branched-chain amino acid (BCAA) concentrations tended to be higher (*P* < 0·1) in Gln piglets than in Ala piglets. In 12-d-old piglets, the BC class affected (*P* < 0·05) the concentration of BCAA, the sum of the groups of IAA and KAA in plasma (Table 4). Within piglets supplemented with Gln, those with LBW had higher plasma valine (Val) (*P* = 0·050) and lower Gly (*P* = 0·027) concentrations than NBW piglets. Within the Ala supplementation group, LBW piglets had higher (*P* < 0·05) plasma His, isoleucine (Ile), Leu, Lys, Met, Val, Ala, Asn, serine (Ser), BCAA, GAA, IAA, KAA and total AA concentrations, and a trend towards higher (*P* = 0·052) plasma DAA concentrations compared with NBW piglets.

**Table 4.** Concentration of plasma-free amino acids at age 12 d of low and normal birth weight piglets supplemented with glutamine or alanine starting at age 1 d

	Ala				Gln				ANOVA *P* ||		
	LBW	se	NBW	se	LBW	se	NBW	se	Sup	BC	Interaction
IAA (μmol/l)											
Arg	185·3	15·1	158·3	15·1	192·9	15·2	202·8*	15·2	0·097	0·567	0·223
His	88·9†	6·1	71·8	6·1	90·9	5·8	99·6*	5·8	0·033	0·450	0·025
Ile	116·7†	9·1	82·5	9·1	107·8	8·8	96·1	8·8	0·799	0·006	0·159
Leu	148·2†	7·5	105·9	7·5	133·0	7·2	126·1*	7·2	0·740	< 0·001	0·009
Lys	175·1†	13·8	139·5	13·8	158·8	13·2	169·6‡	13·2	0·605	0·252	0·037
Met	46·7†	3·3	38·4	3·3	42·7	3·2	48·0*	3·2	0·388	0·579	0·015
Val	242·0†	11·4	197·1	11·4	244·4†	10·9	219·0	10·9	0·272	< 0·001	0·274
DAA, μmol/l											
Ala	1301·0†	80·6	1097·4	80·6	671·5*	77·5	724·2*	77·5	< 0·001	0·278	0·070
Asn	94·2†	3·5	79·4	3·5	82·7*	3·5	86·3	3·5	0·511	0·110	0·011
Gln	528·5	33·3	542·4	33·3	640·4*	31·7	694·2*	31·7	< 0·001	0·197	0·442
Gly	933·6	47·6	867·4	47·6	812·1†,‡	45·8	946·2	45·8	0·659	0·411	0·020
Ser	304·1†	17·1	252·1	17·1	270·8	16·3	271·8	16·3	0·685	0·072	0·061
Groups of AA, mmol/l											
BCAA	0·5†	0·02	0·4	0·02	0·5	0·02	0·4‡	0·02	0·509	< 0·001	0·053
DAA	4·7§	0·2	4·2	0·2	3·9*	0·2	4·1	0·2	0·016	0·459	0·044
GAA	4·7†	0·2	4·1	0·2	3·8*	0·2	3·9	0·2	0·003	0·203	0·043
IAA	1·4†	0·04	1·2	0·04	1·2*	0·04	1·2	0·04	0·315	0·006	0·017
KAA	0·3†	0·02	0·25	0·02	0·3	0·02	0·3*	0·02	0·649	0·021	0·012
Total	6·8†	0·2	6·1	0·2	5·8*	0·2	6·0	0·2	0·015	0·164	0·025

Ala, alanine; Asn, asparagine; Gln, glutamine; LBW, low birth weight; NBW, normal birth weight; Sup, supplementation; BC, birth weight class; IAA, indispensable amino acid; DAA, dispensable amino acid; AA, amino acid; BCAA, branched-chain amino acid; GAA, glucogenic amino acid; IAA, indispensable amino acid; KAA, ketogenic amino acid.

Values are least-squares means ± standard error; *n* 12/group. Plasma samples were obtained at 2 h post-oral administration of Gln or Ala and subsequent milk replacer. Only plasma AA influenced by supplementation or BC are shown.

* Different from Ala-supplemented piglets within BC group (*P* < 0·05).

† Different from NBW piglets within supplemental group (*P* < 0·05).

‡ Tend to differ from Ala-supplemented piglets within BC group (*P* < 0·1).

§ Tend to differ from NBW piglets within supplemental group (*P* < 0·1).

|| ANOVA *F* test. The effect experimental run was significant (*P* < 0·01) for plasma Asn, DAA, GAA, IAA and total AA, but the direction of differences did not change.

### Correlation between plasma parameters and liver TAG concentration and body mass

At 5 d of age (online Supplementary Table 2), plasma Leu, Val and BCAA concentrations of Ala-LBW piglets and plasma 1-MH concentrations of Gln-LBW piglets showed correlations with liver TAG concentrations (*P* < 0·05). There were several plasma-free AA concentrations positively correlated with BM of Ala-NBW piglets (*P* < 0·05), with the correlation coefficient higher than 0·58. Only plasma ALT and Car in Ala-LBW piglets and plasma albumin, AST and Cys in Gln-LBW piglets were correlated with BM at 5 d of age. No correlations between plasma parameters and BM were observed in the 5-d-old Gln-NBW piglets. At 12 d (Supplementary Table 3), liver TAG concentrations correlated with plasma *β*-Ala concentration in Ala-LBW piglets, plasma albumin, Citcitrulline (Cit) and GABA concentrations in Ala-NBW piglets, plasma albumin, protein, Asn and threonine (Thr) concentrations in Gln-LBW piglets and plasma 3-MH concentration in Gln-NBW piglets (*P* < 0·05). Piglet BM showed positive correlations with plasma albumin, protein and several free AA concentrations in 12-d-old Ala-LBW piglets (*P* < 0·05), whereas in the Ala-NBW piglets plasma glucose, insulin, protein, urea, Thr and *α*-aminoadipic acid concentrations were correlated with BM (*P* < 0·05). In Gln-LBW piglets, only plasma albumin concentration was positively correlated with BM (*P* = 0·019). In Gln-NBW piglets, plasma albumin concentration was positively and plasma Asn concentration was negatively correlated with BM at 12 d of age (*P* < 0·05).

## Discussion

### Effect of glutamine supplementation on growth

There are few reports on the effects of supplemental Gln on the growth of suckling piglets^(3,10)^. Our study demonstrated that the Gln effect on piglet growth is BC-dependent, that Gln can benefit the growth of neonatal male LBW piglets, suggesting catch-up growth due to Gln supplementation. The daily dosage of Gln used in our study was identical to the report of Wu *et al.* ^(3)^, which showed an increase in BMG (+16 %, until 21 d of age) in Gln-supplemented LBW piglets (birth weight 0·93 ± 0·06 kg). We did not observe an increase of BMG in Gln-LBW piglets, presumably because in our study the application time was shorter (12 *v*. 21 d) and the birth weight of our LBW piglet population was greater (+18·3 %). Gln supplementation has also been reported to improve piglet BMG when given to lactating sows (1 % of diet)^(38)^ or suckling piglets (1 g/kg BM/d, from 7 to 14 d of age)^(10)^. In contrast, piglet BMG was not affected or was even reduced when Gln was given at 2·5 % of the diet to lactating sows^(39)^ or at 2 g/kg BM/d to piglets from 7 to 21 d^(10)^. However, in these reports, piglet birth weight and sex were not separated^(10,38,39)^, or the litter sizes were smaller (10–11 piglets per litter)^(38,39)^, thus complicating direct comparisons to the present study. In human studies, Gln supplementation in LBW infants showed study-dependent effects on growth^(40–42)^. The reason why the Gln effect is so variable is difficult to deduce from the available information, but differences in birth weight, age and dosage amount may play a role. In addition to daily AA supplementation, maternal milk was the only nutrient source for the piglets in our study. Therefore, the increased growth of Gln-LBW piglets might be associated with the greater milk intake as measured by the deuterium oxide method during 11–12 d of age. Although Gln is a precursor of brain neurotransmitters (Glu, aspartate (Asp) and GABA) involved in food intake regulation^(12,43)^ due to the lack of data on Gln, Glu, Asp and GABA concentration in the piglets’ brain, it cannot be determined whether Gln supplementation is involved in the regulation of milk intake. Another conceivable mechanism to explain higher milk intake could be that Gln supplementation improves intestinal development and function, previously shown in weaning or weaned piglets^(9)^, associated with increased gastrointestinal capacity of neonatal piglets. Our observation of a greater milk intake in Gln-LBW piglets at 11–12 d contradicts two previous studies^(3,10)^, which showed that Gln supplementation (1 g/kg BM/d) to suckling piglets at 7–14 or 0–21 d of age did not affect milk intake, which could be due to different measuring methods^(10,34,44)^. Taken together, our observation of a higher milk intake in Gln-supplemented LBW piglets needs to be confirmed in further investigations. There were no significant correlations between plasma glucose, insulin and protein with BM in Gln-supplemented piglets suggesting that plasma metabolites did not play a role in explaining the higher BM in Gln-LBW piglets.

### Effect of glutamine supplementation on lipid metabolism

In line with our findings in 5-d-old Ala-LBW piglets, elevated plasma TAG concentrations have been reported in LBW human infants^(45)^ and neonatal LBW piglets^(46)^ as compared with NBW individuals and have been associated with the subsequent development of diabetes and obesity later in life in humans^(47,48)^. Notably, Gln supplementation seems to normalise the plasma and liver TAG concentrations of 5-d-old LBW piglets in our study. We found about four times greater area and number of hepatic lipid droplets and an increased rate of hepatic lipolysis in adolescent pigs born with LBW than in NBW pigs^(49)^. Furthermore, in the postprandial state, plasma TAG are derived mainly from VLDL secreted by the liver^(19)^.This might also apply to the neonatal LBW piglets in the present study and could explain the higher plasma and liver TAG concentrations in the 5-d-old Ala-LBW piglets. In contrast, Gln-supplemented LBW but not Ala-LBW piglets present higher plasma concentrations of NEFA than NBW littermates but without showing higher plasma and liver TAG than the Gln-NBW piglets. Higher plasma NEFA concentrations, as for example observed in feed restricted young pigs^(50)^, are considered a marker of lipolysis and negative energy balance. In a previous study, we found a lower fat oxidation in adolescent LBW pigs than in NBW littermates^(51)^. It is possible that Gln may normalise TAG levels in plasma and liver by increasing hepatic lipolysis rates and concomitantly increasing plasma NEFA, but what mechanism may be responsible cannot be determined from the available data.

Elevated liver TAG concentration may be associated with increased plasma AST and ALT which are markers of liver injury^(17,18)^. LBW piglets have been shown to have higher plasma AST and ALT activities than NBW piglets in the early days of life^(17,52)^. In our study, the higher liver TAG concentration in 5-d-old Ala-LBW piglets was not associated with plasma ALT or AST activities, which were within the lower range of healthy pigs reported in previous studies^(53)^.

Plasma insulin concentrations were higher in Ala-LBW piglets than in Ala-NBW piglets at 5 and 12 d of age, whereas others reported no difference in plasma insulin concentrations between neonatal low and NBW piglets^(54)^. The lower plasma glucose:insulin ratio in the LBW groups suggest that these piglets require more insulin to transport glucose across the cell membrane which may indicate a lower glucose tolerance^(55,56)^. Nevertheless, it appears that LBW increases the risk of insulin resistance and poor glucose tolerance in older pigs^(54)^. Interestingly, the differences in plasma and liver TAG concentrations in 5-d-old Ala-LBW piglets compared with Gln-LBW or Ala-NBW piglets were not observed at 12 d of age. This might be associated with the compensatory development of perirenal adipose tissue of neonatal LBW piglets^(57)^.

### Effect of glutamine supplementation on amino acid metabolism

The plasma-free AA pool reflects protein turnover which is an important determinant for individual growth^(16)^, and splanchnic tissues are involved in regulating this pool^(58,59)^. However, the intestine of LBW piglets is less mature than of their normal-weight counterparts^(54,60)^. This might play a role for the birth weight-dependent effects of Gln supplementation on piglet plasma-free AA concentrations. As expected, Gln and Ala supplementation increased plasma Gln and Ala concentrations, respectively, in both age classes. Arg has been suggested as an AA limiting the growth of suckling piglets^(61)^. In our study, Gln supplementation resulted in higher plasma Arg concentrations in piglets, which might be explained by the role of Gln as a precursor of endogenous synthesis of Cit for Arg synthesis^(62)^. At 12 d of age, the reduced concentrations of several proteinogenic AA (e.g. His, Ile and Met) in Ala-NBW *v*. Gln-NBW suggested that Gln supplementation seemed to maintain the plasma-free AA pool of NBW piglets, which might be related to the increased milk intake in Gln-NBW piglets and/or supplemental Gln as Gln is also a precursor of other AA such as Cit and Arg^(3)^. Interestingly, the increased milk intake in 12-d-old Gln-LBW piglets did not lead to higher plasma AA concentrations. This could be explained by the higher plasma urea concentrations in Gln-supplemented piglets at 12 d of age, which indicated higher AA oxidation^(63,64)^ as Gln has a carrier function to transport ammonia to the liver urea synthesis^(65)^. Car is mainly synthesised and stored in skeletal muscle and its synthesis is limited by the availability of *β*-Ala^(66)^ which is mainly produced from pyrimidine catabolism and Car hydrolysis^(67)^. However, in our study, neither AA supplementation nor BC affected plasma *β*-Ala concentrations, as well as the intramuscular *β*-Ala and Car concentrations as shown in a companion study^(68)^. The lower plasma Car concentrations in 5-d-old Ala-LBW piglets *v*. Ala-NBW and Gln-LBW could be associated with muscle metabolism or function, which requires further investigations.

Our findings are partly consistent with other studies which reported modest or no effect of Gln supplementation on plasma AA concentrations in LBW human infants^(69,70)^, whereas the effect on plasma urea concentrations differed between studies^(42,69)^. The differing observations in previous studies might be due to the differences in birth weight (0·5–2·5 kg), Gln dosage (0·3–1·25 g/kg BM/d) and age (3–74 d of age)^(69,70)^. Because the intestine plays an important role in AA absorption, catabolism and synthesis, the observed plasma-free AA concentrations might indicate a potential effect of oral Gln supplementation on intestinal function and AA metabolism, and the different observations at 5 and 12 d could be associated with developmental changes in intestinal AA metabolism^(71)^.

In conclusion, the effect of supplemental Gln in neonatal piglets was birth weight-dependent. We found Gln supplementation associated with improved growth and altered lipid metabolism in LBW piglets and plasma-free AA profiles in NBW piglets. These effects were associated with higher milk intake and suggest potential effects of Gln supplementation on liver, muscle and intestinal function, which warrants further investigations.

## Supplementary material

For supplementary material accompanying this paper visit https://doi.org/10.1017/S0007114522000459. click here to view supplementary material
