# Supplementary material for: Glutamine supplementation moderately affects growth, plasma metabolite and free amino acid patterns in neonatal low birth weight piglets
Source: Br J Nutr. 2022 Feb 11;128(12):2330–40. doi: 10.1017/S0007114522000459 (PMC9723486; doi:10.1017/S0007114522000459)
Supplement: Supplementary file 1 [file S0007114522000459sup001.docx]

**Supplementary Table 1** Concentration of sows’ colostrum and milk macronutrients, immunoglobulins and amino acids until day 12 of lactation

| Stage of lactation | 2 hours | | | 24 hours | | 7 days | | 12 days | | | ANOVA *p* values^2^ |
| --- | --- | --- | --- | --- | --- | --- | --- | --- | --- | --- | --- |
|  | LSM | SE | LSM | | SE | LSM | SE | | LSM | SE | Stage of lactation |
| Dry matter, % | 27.95^a^ | 0.62 | 25.20^b^ | | 0.65 | 21.66^c^ | 0.61 | | 22.21^c^ | 0.60 | < 0.001 |
| Crude protein, % | 17.54^a^ | 0.48 | 9.23^b^ | | 0.50 | 5.56^c^ | 0.47 | | 5.61^c^ | 0.46 | < 0.001 |
| Crude fat, % | 6.84^b^ | 0.42 | 11.20^a^ | | 0.44 | 9.56^a^* | 0.41 | | 10.65^a^ | 0.40 | < 0.001 |
| Lactose, % | 1.51^c^ | 0.12 | 2.86^b^ | | 0.12 | 4.66^a^ | 0.12 | | 4.39^a^ | 0.11 | < 0.001 |
| IgG, mg/mL | 34.07^a^ | 1.37 | 8.73^b^ | | 1.44 | 1.69^c^ | 1.35 | | 1.16^c^ | 1.32 | < 0.001 |
| IgA, mg/mL | 16.01^a^ | 1.40 | 6.96^b^ | | 1.48 | 2.76^b^ | 1.38 | | 3.46^b^ | 1.36 | < 0.001 |
| IgM, mg/mL | 8.29^a^ | 0.35 | 4.33^b^ | | 0.37 | 1.81^c^ | 0.35 | | 1.78^c^ | 0.34 | < 0.001 |
| Gln |  |  |  | |  |  |  | |  |  |  |
| Free, mg/L | 2.54^b^ | 9.19 | 9.49^b^ | | 9.68 | 99.84^a^ | 9.05 | | 82.46^a^ | 8.87 | < 0.001 |
| Protein bound, g/L | 12.75^a^ | 0.37 | 6.84^b^ | | 0.42 | 4.95^c^ | 0.37 | | 4.97^c^ | 0.36 | < 0.001 |
| Total, g/L | 12.75^a^ | 0.37 | 6.85^b^ | | 0.42 | 5.05^c^ | 0.37 | | 5.05^c^ | 0.36 | < 0.001 |
| Glu |  |  |  | |  |  |  | |  |  |  |
| Free, mg/L | 7.25^b^ | 6.93 | 26.61^b^ | | 7.30 | 107.26^a^ | 6.82 | | 88.36^a^ | 6.69 | < 0.001 |
| Protein bound, g/L | 13.30^a^ | 0.38 | 7.80^b^ | | 0.43 | 4.54^c^ | 0.38 | | 4.42^c^ | 0.37 | < 0.001 |
| Total, g/L | 13.30^a^ | 0.38 | 7.83^b^ | | 0.42 | 4.65^c^ | 0.38 | | 4.51^c^ | 0.37 | < 0.001 |
| Ala |  |  |  | |  |  |  | |  |  |  |
| Free, mg/L | 2.50^c^ | 2.52 | 5.54^c^ | | 2.66 | 40.58^a^ | 2.49 | | 27.82^b^ | 2.44 | < 0.001 |
| Protein bound, g/L | 6.38^a^ | 0.18 | 3.22^b^ | | 0.21 | 1.55^c^ | 0.18 | | 1.56^c^ | 0.18 | < 0.001 |
| Total, g/L | 6.38^a^ | 0.18 | 3.22^b^ | | 0.21 | 1.59^c^ | 0.18 | | 1.58^c^ | 0.18 | < 0.001 |
| Asn |  |  |  | |  |  |  | |  |  |  |
| Free, mg/L | 0.61^b^ | 0.40 | 1.37^b^ | | 0.42 | 5.81^a^ | 0.39 | | 4.47* | 0.38 | < 0.001 |
| Protein bound, g/L | 4.85^a^ | 0.13 | 2.77^b^ | | 0.14 | 1.65^c^ | 0.13 | | 1.63^c^ | 0.12 | < 0.001 |
| Total, g/L | 4.85^a^ | 0.13 | 2.77^b^ | | 0.14 | 1.65^c^ | 0.13 | | 1.63^c^ | 0.12 | < 0.001 |
| Asp |  |  |  | |  |  |  | |  |  |  |
| Free, mg/L | 1.74^c^ | 2.89 | 2.34^c^ | | 3.04 | 39.42^a^ | 2.84 | | 22.36^b^ | 2.79 | < 0.001 |
| Protein bound, g/L | 6.62^a^ | 0.17 | 3.93^b^ | | 0.19 | 2.08^c^ | 0.17 | | 2.04^c^ | 0.17 | < 0.001 |
| Total, g/L | 6.63^a^ | 0.17 | 3.93^b^ | | 0.19 | 2.12^c^ | 0.17 | | 2.07^c^ | 0.17 | < 0.001 |
| All amino acids |  |  |  | |  |  |  | |  |  |  |
| Free, mg/L | 78.48^c^ | 30.34 | 196.89^b^ | | 31.97 | 611.94^a^ | 29.88 | | 509.59* | 29.29 | < 0.001 |
| Protein bound, g/L | 148.50^a^ | 3.95 | 78.98^b^ | | 4.42 | 45.74^c^ | 3.92 | | 45.87^c^ | 3.84 | < 0.001 |
| Total, g/L | 148.56^a^ | 3.95 | 79.06^b^ | | 4.42 | 46.17^c^ | 3.92 | | 46.22^c^ | 3.84 | < 0.001 |

Values are least-squares means ± standard error. Colostrum/milk proximate composition, immunoglobulin and free AA concentrations: *n* = 8 (2 h, 24 h, 7 d, 12 d); protein bound and total AA concentrations: *n* = 8 (2 h, 7 d, 12 d), *n* = 7 (24 h).

^a-c^ Labeled values without a common letter differ from the neighboring lactation stages (*P* < 0.05, Tukey-Kramer test).

* Labeled values tend to differ from the previous neighboring lactation stage (*P* < 0.1, Tukey-Kramer test).

**Supplementary Table 2** Pearson correlations between plasma free amino acid, metabolite, insulin and liver triglyceride concentrations or body mass of 5-d-old low and normal birthweight piglets supplemented with glutamine or alanine starting at age 1 d^1^

|  | Ala-LBW | | | Ala-NBW | | Gln-LBW | | Gln-NBW | | |
| --- | --- | --- | --- | --- | --- | --- | --- | --- | --- | --- |
|  | *r* | *p* | *r* | | *p* | *r* | *p* | | *r* | *p* |
| Plasma parameters – liver triglycerides | | | | | | | | | | |
| Leu | 0.60 | 0.049 | 0.37 | | 0.235 | -0.47 | 0.171 | | -0.37 | 0.236 |
| Val | 0.62 | 0.040 | 0.22 | | 0.500 | -0.48 | 0.156 | | -0.22 | 0.495 |
| 1-MH | 0.10 | 0.770 | -0.27 | | 0.392 | -0.74 | 0.015 | | 0.47 | 0.123 |
| BCAA | 0.63 | 0.037 | 0.37 | | 0.233 | -0.38 | 0.277 | | -0.29 | 0.357 |
| Plasma parameters – body mass | | | | | | | | | | |
| Albumin | 0.37 | 0.262 | 0.39 | | 0.208 | 0.66 | 0.038 | | 0.46 | 0.128 |
| AST | 0.14 | 0.672 | -0.14 | | 0.669 | -0.67 | 0.034 | | 0.43 | 0.162 |
| ALT | -0.64 | 0.035 | -0.51 | | 0.089 | -0.47 | 0.175 | | -0.38 | 0.224 |
| Cys | 0.48 | 0.134 | 0.79 | | 0.002 | 0.68 | 0.030 | | 0.03 | 0.921 |
| Gly | 0.21 | 0.531 | 0.61 | | 0.035 | 0.33 | 0.359 | | 0.20 | 0.533 |
| Orn | 0.36 | 0.274 | 0.71 | | 0.010 | 0.46 | 0.178 | | -0.25 | 0.443 |
| Pro | 0.45 | 0.165 | 0.85 | | 0.000 | -0.14 | 0.699 | | -0.19 | 0.555 |
| Ser | 0.47 | 0.148 | 0.73 | | 0.007 | -0.07 | 0.851 | | -0.24 | 0.452 |
| Tau | 0.16 | 0.632 | 0.62 | | 0.031 | -0.19 | 0.606 | | -0.14 | 0.664 |
| Thr | 0.51 | 0.113 | 0.59 | | 0.045 | 0.14 | 0.708 | | -0.45 | 0.141 |
| Trp | 0.34 | 0.305 | 0.59 | | 0.044 | -0.06 | 0.867 | | -0.08 | 0.794 |
| Val | 0.27 | 0.428 | 0.68 | | 0.015 | -0.09 | 0.804 | | -0.33 | 0.290 |
| Anser | 0.05 | 0.881 | 0.58 | | 0.050 | 0.29 | 0.419 | | -0.49 | 0.105 |
| Car | 0.70 | 0.016 | 0.27 | | 0.400 | -0.41 | 0.234 | | 0.12 | 0.699 |
| AFAA | 0.51 | 0.108 | 0.78 | | 0.003 | -0.12 | 0.748 | | -0.27 | 0.387 |
| BCAA | 0.29 | 0.380 | 0.59 | | 0.045 | -0.19 | 0.593 | | -0.30 | 0.342 |
| DAA | 0.60 | 0.053 | 0.80 | | 0.002 | -0.18 | 0.624 | | -0.28 | 0.384 |
| GFAA | 0.51 | 0.112 | 0.77 | | 0.003 | -0.14 | 0.699 | | -0.28 | 0.383 |
| IAA | 0.49 | 0.122 | 0.66 | | 0.021 | -0.12 | 0.749 | | -0.36 | 0.257 |
| Total | 0.59 | 0.058 | 0.86 | | 0.000 | -0.11 | 0.769 | | -0.29 | 0.356 |

^1^ AFAA, arginine family amino acids; Anser, Anserine; BCAA, branched-chain amino acid; DAA, dispensable amino acid; GFAA, glutamate family amino acids; IAA, indispensable amino acid; 1-MH, 1-methylhistidine. *n* = 12/group. Only plasma parameters showing significant correlation with liver triglyceride concentration or body mass are presented.

**Supplementary Table 3** Pearson correlations between plasma free amino acid, metabolite, insulin and liver triglyceride concentrations or body mass of 12-d-old low and normal birthweight piglets supplemented with glutamine or alanine starting at age 1 d^1^

|  | Ala-LBW | | | Ala-NBW | | Gln-LBW | | Gln-NBW | | |
| --- | --- | --- | --- | --- | --- | --- | --- | --- | --- | --- |
|  | *r* | *p* | *r* | | *p* | *r* | *p* | | *r* | *p* |
| Plasma parameters – liver triglycerides | | | | | | | | | | |
| Albumin | 0.07 | 0.820 | -0.65 | | 0.021 | -0.63 | 0.028 | | -0.20 | 0.523 |
| Protein | 0.08 | 0.812 | -0.47 | | 0.120 | -0.59 | 0.044 | | 0.33 | 0.302 |
| Asn | -0.31 | 0.326 | 0.20 | | 0.525 | 0.64 | 0.024 | | 0.19 | 0.558 |
| Cit | -0.03 | 0.928 | 0.79 | | 0.002 | 0.35 | 0.265 | | 0.19 | 0.545 |
| Thr | 0.45 | 0.144 | 0.22 | | 0.493 | 0.69 | 0.012 | | 0.15 | 0.643 |
| β-Ala | -0.58 | 0.047 | 0.34 | | 0.286 | -0.11 | 0.736 | | 0.00 | 0.995 |
| 3-MH | 0.10 | 0.746 | -0.14 | | 0.664 | -0.27 | 0.391 | | -0.63 | 0.027 |
| GABA | 0.08 | 0.807 | -0.66 | | 0.020 | 0.29 | 0.360 | | 0.19 | 0.551 |
| Plasma parameters – body mass | | | | | | | | | | |
| Albumin | 0.70 | 0.012 | 0.48 | | 0.111 | 0.66 | 0.019 | | 0.60 | 0.040 |
| Glucose | 0.36 | 0.248 | 0.70 | | 0.012 | 0.48 | 0.114 | | 0.23 | 0.470 |
| Insulin | -0.01 | 0.967 | 0.68 | | 0.015 | 0.49 | 0.104 | | -0.29 | 0.360 |
| Protein | 0.65 | 0.023 | 0.72 | | 0.008 | 0.53 | 0.073 | | 0.35 | 0.264 |
| Urea | 0.57 | 0.053 | 0.76 | | 0.005 | 0.06 | 0.861 | | -0.08 | 0.801 |
| Arg | 0.67 | 0.016 | 0.18 | | 0.576 | -0.19 | 0.556 | | 0.27 | 0.394 |
| Asn | 0.01 | 0.972 | -0.36 | | 0.253 | -0.51 | 0.091 | | -0.60 | 0.040 |
| Cit | 0.66 | 0.019 | -0.33 | | 0.296 | -0.48 | 0.114 | | 0.11 | 0.735 |
| Orn | 0.61 | 0.036 | 0.05 | | 0.883 | -0.39 | 0.206 | | 0.17 | 0.596 |
| Thr | 0.03 | 0.934 | -0.62 | | 0.032 | -0.46 | 0.134 | | -0.18 | 0.586 |
| Trp | 0.82 | 0.001 | 0.44 | | 0.150 | 0.04 | 0.901 | | 0.34 | 0.273 |
| Tau | 0.58 | 0.047 | 0.27 | | 0.396 | -0.39 | 0.210 | | -0.18 | 0.571 |
| AAAP | -0.32 | 0.319 | 0.64 | | 0.025 | -0.03 | 0.933 | | -0.43 | 0.164 |

^1^ AAAP, α-aminoadipic acid; GABA, γ-Aminobutyric acid; 3-MH, 3-methylhistidine. *n* = 12/group. Only plasma parameters showing significant correlation with liver triglyceride concentration or body mass are presented.
